# High-throughput screening for texturing *Lactococcus* strains

**DOI:** 10.1093/femsle/fnz001

**Published:** 2019-01-09

**Authors:** Vera Kuzina Poulsen, Patrick Derkx, Gunnar Oregaard

**Affiliations:** Discovery, R&D, Chr. Hansen A/S, 10–12 Bøge Allé, DK2970, Hørsholm, Denmark

**Keywords:** polysaccharide, texture, *Lactococcus*, screening

## Abstract

In the food industry, lactic acid bacteria (LAB) are used in dairy fermentations, extending the shelf life by lowering the pH and also affecting taste and texture of the fermented milk. The texture of fermented milk is an important quality parameter, affecting consumer acceptance. Finding LAB providing desired texture of a product is time consuming and laborious when using standard methods for measuring texture, e.g. rheology measurements. Screening of 986 *Lactococcus lactis* strains resulted in few strains with the ability to enhance texture, demonstrating the necessity of implementation of high-throughput screening methods. A high-throughput screening assay was developed, combining small-scale 96-well microtiter plates and pressure measurements during liquid handling, e.g. aspiration, to find strains that give good texture in fermented milk. Only about 1% of the strains were found to enhance milk texture. Two of the texturing strains belong to *L. lactis* subsp. *lactis*, which are the first texturing strains from this subsp. reported. Mining for *eps* gene clusters responsible for exocellular polysaccharide production was performed, as polysaccharide production can contribute positively to fermented milk texture. Comparative genomics approach revealed four types of texturing *L. lactis* strains with diverse *eps* gene clusters.

## Teaser

Novel method for high-throughput screening for texturing *Lactococcus* strains and comparative genomics of gene clusters for polysaccharide production.

## INTRODUCTION

Conversion of fresh milk to fermented milk by lactic acid bacteria (LAB) is used to extend the shelf life of milk due to acidification, to provide taste as well as texture. The ability of LAB to produce polysaccharides is associated with improvement of the texturing properties and increased health benefits of fermented products (Caggianiello, Kleerebezem and Spano 2016). Polysaccharides from food-grade LAB are non-toxic, biodegradable, environment friendly and act as natural viscosifiers, emulsifiers, stabilisers, binders, gelling agents, coagulants and suspending agents in food industry as well as in cosmetics (Jindal and Singh Khattar 2018). For this reason, screening and selection of polysaccharide-producing LAB is of importance to both academia and industry.

*Lactococcus lactis* is used to produce numerous fermented dairy products including cheese and mesophilic fermented milk, such as buttermilk and sour cream. Polysaccharide-producing strains are of great interest for these applications, as polysaccharides released into the medium can result in improved texturing properties of buttermilk and sour cream, while capsular polysaccharides can result in improved water-holding capacity and thus improved yields of cheese.

Genes encoding Wzy-dependent exocellular polysaccharide biosynthesis proteins in LAB are typically organised in a cluster with an operon structure. The major genera of LAB used in food processing (*Lactococcus, Streptococcus, Lactobacillus, Leuconostoc, Oenococcus* and *Pediococcus*) possess *eps* gene clusters (Zeidan *et al.*^2017^). Generally, *eps* gene clusters are highly diverse and their nucleotide sequences are among the most variable sequences in LAB genomes. Also, polysaccharides structures are very diverse as a result of variations in monosaccharide building blocks, the presence of branches, decoration with non-carbohydrate moieties and linkages: for instance, two glucose residues can be joined together in 30 different ways (Laine 1994, Zeidan *et al*. 2017).

The type and size of polysaccharides and their interaction with milk proteins are the determining factors for texture development (Hassan 2008; Mende, Rohm and Jaros 2016; Birch *et al.*^2017^). Even though the potential to synthesise polysaccharides is encoded within the genomes of many LAB, production of polysaccharides and their functional properties need to be evaluated for successful industrial applications.

A great variety of different techniques to identify polysaccharide-producing microorganisms have been devised. Screening strategies can be generally divided into screening for polysaccharide-producing strains or phenotypes associated with these polysaccharides. They include visual inspection of slimy or mucoid colonies on solidified media, observation of viscosity in culture broth (e.g. measuring ropiness with a pipette or by microhaematocrit capillaries), staining methods (aniline blue, ruthenium red, neutral red, Calcofluor white, Congo red, Indian ink, lectins), carbohydrate determination (quantitative HPLC analysis, total sugar determination using phenol-sulphuric acid method), cell sedimentation in semi-liquid agar medium, measurement of the change in the electrical conductivity in the growth medium during polysaccharide production (impedance microbiology), precipitation with different alcohols as a common detection and isolation method, electron microscopy and FITC-dextran exclusion assay for visualisation of the capsule production (Llull *et al.*^2005^; Hathaway *et al.*^2012^; Rühmann, Schmid and Sieber 2015; Zeidan *et al.*^2017^; Bancalari *et al.*^2018^).

The texture of fermented milk is dependent on both the bacteria used for fermentation and process parameters. Polysaccharide-producing bacteria can positively influence product characteristics such as texture and sensory properties (Mende, Rohm and Jaros 2016). Rheometer and texture analyser are typically used to assess texturing properties of milk gels, such as shear stress. Sensory textural attributes are often correlated with the results from instrumental tests, e.g. shear stress is related to viscosity and perceived mouth thickness (Folkenberg *et al.*^2006^). Standard texture measurements require relatively large sample volumes, e.g. 100 ml. Although these methods of measurement are accurate and reproducible, they are highly demanding in respect of the time required per sample, technical skills and precision. The state of the art equipment for measuring viscosity takes about 20 min per sample, and only one sample at a time can be tested. The washing and changing step between each sample makes it difficult to be used in a high-throughput screening method. There is a need for high-throughput screening methods that may be used for relevant food matrices such as acidified milk.

The aim of this work was to develop a method for high-throughput texture screening of fermented milk samples and screen a collection of 986 *Lactococcus* strains for texturing capabilities. Moreover, we wanted to examine if any of the texturing strains found had novel *eps* gene clusters, compared to what has previously been described in the literature.

## MATERIALS AND METHODS

### High-throughput screening for texturing strains

**Figure 1.**
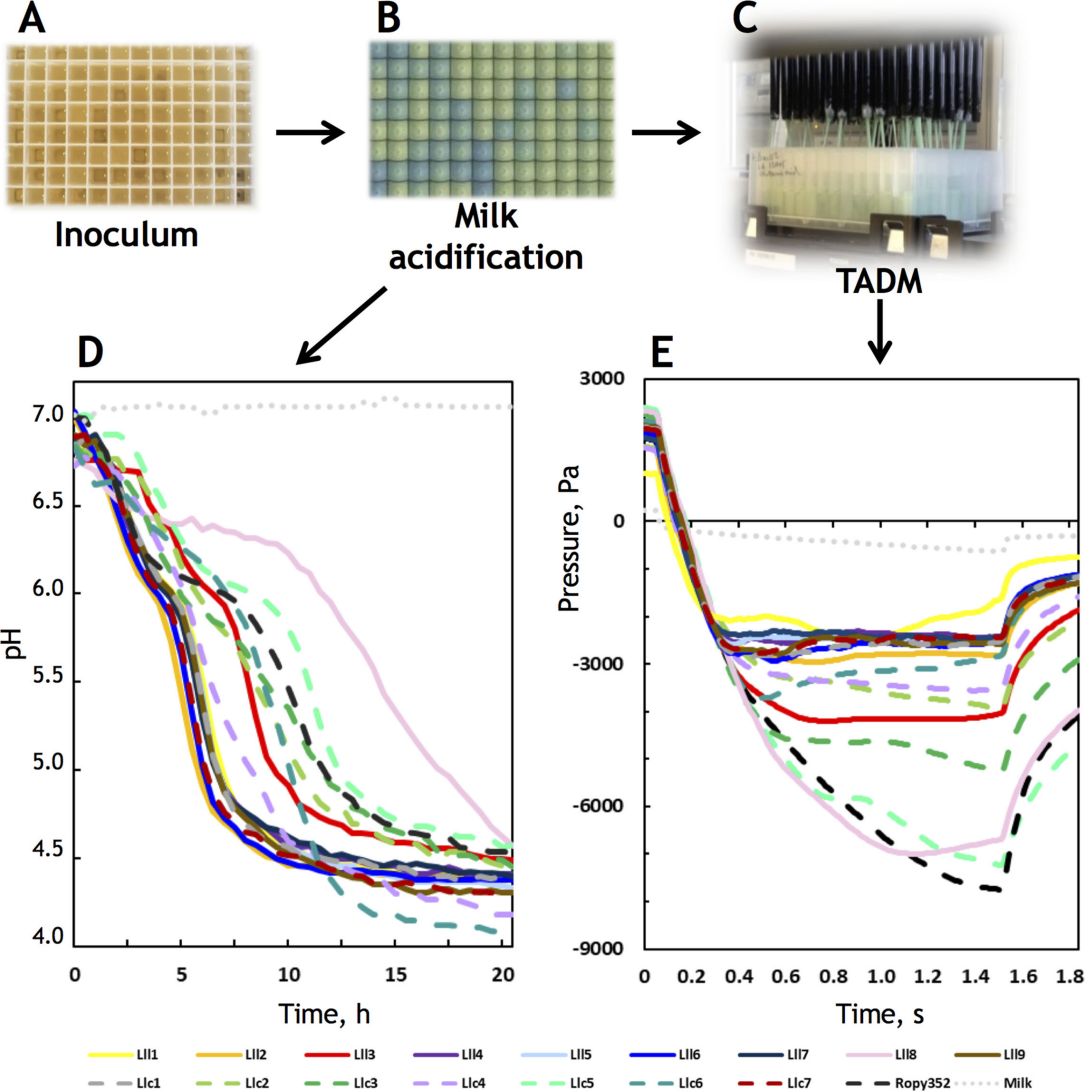
Experimental setup for characterisation of strains for their milk acidification and texturing properties in 96-well microtiter plate format. Overnight cultures in M17 (**A**) were transferred to milk. Milk with pH indicator was fermented in microtiter plates, which were incubated on flat-bed scanners at 30°C (**B**). The bottom of the plates was scanned every 6 min, and the colour development was correlated with pH changes in milk. The milk acidification results for selected strains are shown in (**D**). The texturing properties of the strains were assessed using TADM tool of the Hamilton liquid handling robot. Pressure versus time curves of selected strains obtained during pipetting of the samples are shown in (**E**). Dashed lines (**D** and **E**) represent *L. lactis* subsp. *cremoris* strains and the non-acidified (not inoculated) milk sample. Strains in D and E represent all the proprietary *L. lactis* texturing strains found during the screening together with a few representatives of non-texturing strains, for which genome sequences were available.

A total of 986 *L. lactis* strains originating from the Chr. Hansen culture collection were screened for ability to enhance texture in fermented milk. The strains were inoculated in 96 low-well microtiter plates in 200 μl M17 broth (Terzaghi and Sandine 1975) containing 1% glucose and 1% lactose as C-source and incubated overnight at 30°C. A volume of 10 μl was transferred to 990 μl B-milk containing pH colour indicator ±0.2% yeast extract in 96 deep-well plates. B-milk was prepared by reconstituting low fat skim milk powder to a level of dry matter of 9.5% and pasteurised at 99°C for 30 min, followed by cooling to 30°C. The pH colour indicator milk was prepared in the following way: 50 mg bromocresol purple salt and 50 mg bromocresol green salt (both from Sigma Aldrich, St. Louis, Missouri, United States) were dissolved in a final volume of 40 ml dH_2_O, pH was adjusted to 7.0 with NaOH and the final volume was adjusted to 50 ml with dH_2_O. The pH indicator was sterile filtered (0.2 μm) and 5 ml was added to 95 ml B-milk. The inoculated pH colour indicator milk samples were incubated for 18–20 h on top of flat-bed scanners (HP ScanJet G4010) with temperature-controlled hoods set at 30°C (Fig. 1). pH-dependent changes in colour were recorded every 6 min, using *pH Multiscan* software (v.5.1, HNH Consult Aps, 9530 Støvring, Denmark). After 18-h static incubation at 30°C, most samples in the plate had a pH of 4.3–4.5, where the fresh milk was converted to fermented milk gel. The plates were kept at 4°C overnight, and TADM (Total Aspiration Dispense Monitoring) pressure curves were obtained by aspirating the samples using Hamilton liquid robot.

A Hamilton MicroLab Star liquid handling device (Hamilton, Bonaduz, Switzerland) was used to collect pressure versus time data using TADM software of the Hamilton (Camenisch 2001). Aspiration pressure curves were used to identify samples with elevated texture. A volume of 500 μl was aspirated (350 μl/s) using wide-bore tips (Hamilton Robotics). Pressure versus time data (TADM) were expressed as a single number by either recording pressure at a particular time point (e.g. 1 s) or as TADM curve area.

Three different tools for carbohydrate active enzyme annotation in dbCAN2 (http://cys.bios.niu.edu/dbCAN2) were combined to classify glucosyltransferases: HMMER search against the dbCAN HMM (hidden Markov model) database, DIAMOND search against the CAZy pre-annotated CAZyme sequence database and Hotpep search against the conserved CAZyme short peptide database (Zhang, Yohe and Huang 2018).

*Lactococcus lactis* subsp. *lactis* strain Lll3 (CHCC11848) was deposited with DSMZ-Deutsche Sammlung von Mikroorganismen und Zellkulturen GmbH, Inhoffenstr. 7B, D-38124 Braunschweig, on 21 August 2014 under the accession no. DSM 29291.

### Rheology measurements

Shear stress data were obtained by inoculating strains in semi-fat milk (1.5% fat) enriched with 2% skim milk powder. Milk was heated at 90°C for 20 min and cooled down to the inoculation temperature, prior to inoculation with 1% overnight microbial culture. The inoculation took place for 7–20 h at 30°C in 200-ml scale until pH = 4.55, as monitored by pH electrodes, followed by cooling to 4°C and storage for 5 days at 4°C. After storage, the fermented milk was manually stirred gently by means of a stick fitted with a bored disc until homogeneity of the sample. Shear stress of the samples was assessed on a rheometer (Anton Paar Physica Rheometer with ASC, Automatic Sample Changer, Anton Paar^®^ GmbH, Austria). For the data analysis, the shear stress at shear rate 300 s^−1^ was chosen. Rheology measurements were made on duplicate biological samples.

### Genome data used for *eps* gene cluster mining

Mining for *eps* gene clusters was performed using BLAST analysis of the conserved part of the lactococcal *eps* gene clusters, against the proprietary genomes and genomes available on the NCBI website. Proprietary genomes were obtained by the purification of total DNA from a culture grown overnight at 30°C in M17 medium containing 1% lactose and 1% glucose using DNeasy Blood and Tissue Kit (Qiagen). The DNA quality was checked using gel electrophoresis, the DNA concentration was estimated using Nanodrop 2000 spectrophotometer and 15 μg DNA (approximately 150 ng/μl) was used for sequencing at BGI (HongKong, China) using Illumina HiSeq equipment with pair-end channel module with 2 × 100 bp read length and an insert size of 500 bp. Assembly of raw data into contigs was performed using CLC Genomics workbench 7.0 software.

Easyfig software (version 2.1) was used for comparative *eps* gene cluster visualisation (Sullivan, Petty and Beatson 2011). In order to compare the most related *eps* gene clusters between each other in Easyfig, the relatedness of the *eps* gene clusters on nucleotide level was assessed using ‘Create Tree’ tool within ‘Alignments and Trees’ set of tools of the CLC Main Workbench 7 software (tree construction method: neighbour joining; nucleotide distance measure: Jukes–Cantor; bootstrapping was performed using 200 replicates).

*Eps* gene cluster sequences obtained from proprietary strains were deposited in GenBank under the following accession numbers: Lll1, MH678623; Lll2, MH678624; Lll3, MH678625; Lll4, MH678626; Lll5, MH678627; Lll6, MH678629; Lll7, MH678630; Lll8, MH678633; Llc1, MH678628; Llc2, MH678632; Llc3, MH678635; Llc4, MH678634; Llc5, MH678631; Llc6, MH678636.

## RESULTS AND DISCUSSION

### High-throughput screening for texturing strains

Milk samples fermented with 986 *Lactococcus* strains were screened for texture using the Hamilton Microlab Star robot. Most strains show similar pressure curves, as represented by strains Lll1, Llc1, Lll4, Lll5, Lll6, Lll7, Llc7 and Lll9 (Fig. 1E). Only eight strains had pressure curves that went deeper: Lll3, Lll8, Llc2, Llc3, Llc4, Llc5, Llc6 and Ropy352 (Fig. 1E). In the milk type used (B-milk), strains with TADM pressure at 1 s below –3000 Pa were considered texturing.

To confirm acidification and texturing abilities of selected strains in a larger scale, the strains were inoculated in 200 ml milk until pH = 4.5, followed by rheometer measurements. The acidification of milk in 200-ml scale was followed using pH electrodes; the acidification curves resembled those obtained in 1-ml scale for the strains under investigation (data not shown).

**Figure 2.**
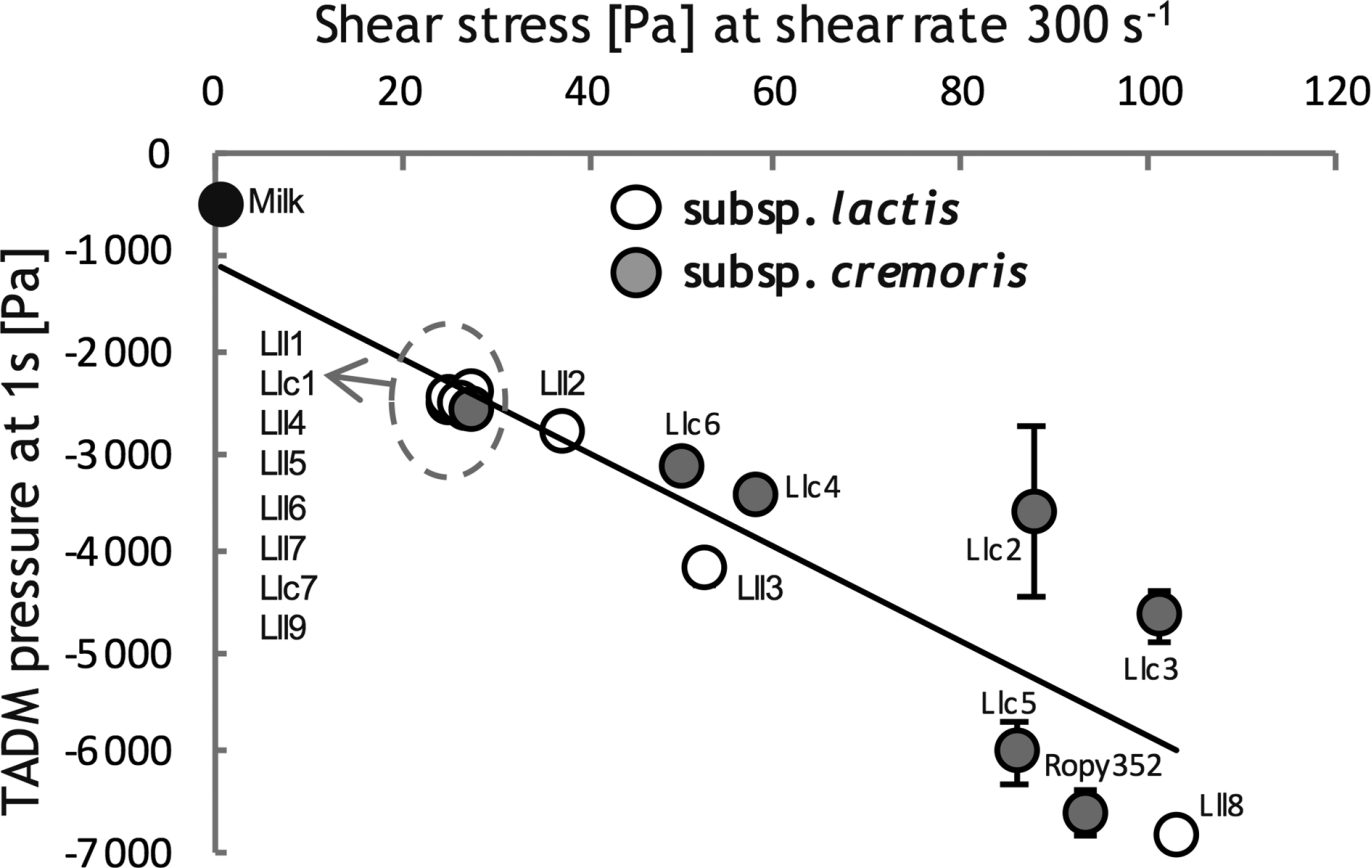
Aspiration pressure values at 1 s (Pa, mean and its standard error, two to eight biological replicates in one to two independent experiments) measured by pipetting using Hamilton liquid handling unit plotted against shear stress (Pa, mean and its standard error, two to four biological replicates in one to two independent experiments) at shear rate 300 s^−1^ values measured using rheometer for selected milk gel samples obtained by fermenting milk using *L. lactis* strains as in Fig. 1. Grey symbols represent *L. lactis* subsp. *cremoris* strains (named Llc1–7), while the white symbols represent *L. lactis* subsp. *lactis* strains (named Lll1–9). ‘Milk’ refers to non-acidified B-milk (not inoculated).

TADM data obtained from 1-ml scale milk acidification were plotted against shear stress values for the same strains from 200-ml scale milk acidification experiment, where the correlation of R^2 ^= 0.82 between TADM data and shear stress measurements was observed (Fig. 2). Shear stress measurements were used to differentiate between texturing and non-texturing strains. Strains resulting in shear stress values below 50 Pa were considered non-texturing, while those above 50 Pa texturing, based on sensory analysis of food-grade fermented milk samples (data not shown). Time to pH 4.5 was between 9.0 and 13.0 h in the case of non-texturing, and 12–20.0 h in the case of texturing strains. Most of the texturing strains belong to *L. lactis* subsp. *cremoris*. To the best of our knowledge, Lll3 is the first texturing strain belonging to *L. lactis* subsp. *lactis* reported (Poulsen, Oeregaard and Derkx 2015). Lll3 and Lll8 are the only texturing strains belonging to the subsp. *lactis* reported so far.

Using the 96 channel pipetting head of the Hamilton robot, 96 samples were examined in a few seconds, while this would take ∼32 h non-stop using a conventional rheometer. The high-throughput method for texture screening in fermented milk can be used to screen thousands of samples within a few hours.

### Comparative genomics of polysaccharide biosynthesis in *L. lactis*

Since an enhanced texture is associated with the production of polysaccharides, mining for *eps* gene clusters was performed. The *eps* gene clusters are generally chromosomal in *L. lactis* subsp. *lactis* but can reside on a plasmid in *L. lactis* subsp. *cremoris* (Table 1).

**Table 1.** List of *L. lactis* strains containing *eps* gene clusters.

Spp.	Strain	*eps* cluster localisation	GenBank nr	Origin of strain
*Lactococcus lactis* subspecies *lactis*				
	AI06	Chromosome	CP009472	Plant
	YF11	Chromosome	APAV00000000	Dairy
	G423	Chromosome	CP024958	Medium (growth broth)
	F44	Chromosome	CP024954	Medium (growth broth)
	KF147	Chromosome	CP001834	Plant
	NCDO 2118	Chromosome	CP009054	Plant
	KLDS 4.0325	Chromosome	CP006766	Dairy
	275 (p275B)	Plasmid	CP016700	Dairy
	UC11	Chromosome	CP015904	Fermented meat
	UC08	Chromosome	CP015903	Fermented meat
	CNCM I-1631	Chromosome	AGHX00000000	Dairy
	229 (p229E)	Plasmid	CP016698	Dairy
	1AA59	Chromosome	AZQT00000000	Dairy
	S0	Chromosome	CP010050	
*Lactococcus lactis* subspecies *cremoris*				
	A76	Chromosome	CP003132	Dairy
	Ropy352 (pEps352)	Plasmid	EF192213	Dairy
	HO2 (pCl658)	Plasmid	AF142639	Dairy
	JM3 (pJM3C)	Plasmid	CP016739	Dairy
	NIZO B40 (pNZ4000)	Plasmid	AF036485	Dairy
	SMQ-461	Chromosome	AY741550	Dairy
	JM2	Chromosome	CP015900	Dairy
*Lactococcus lactis* subspecies *lactis* biovar *diacetylactis*				
	FM03	Chromosome	CP020604	Dairy

The genome data were collected from Genbank on 4 April 2018.

**Figure 3.**
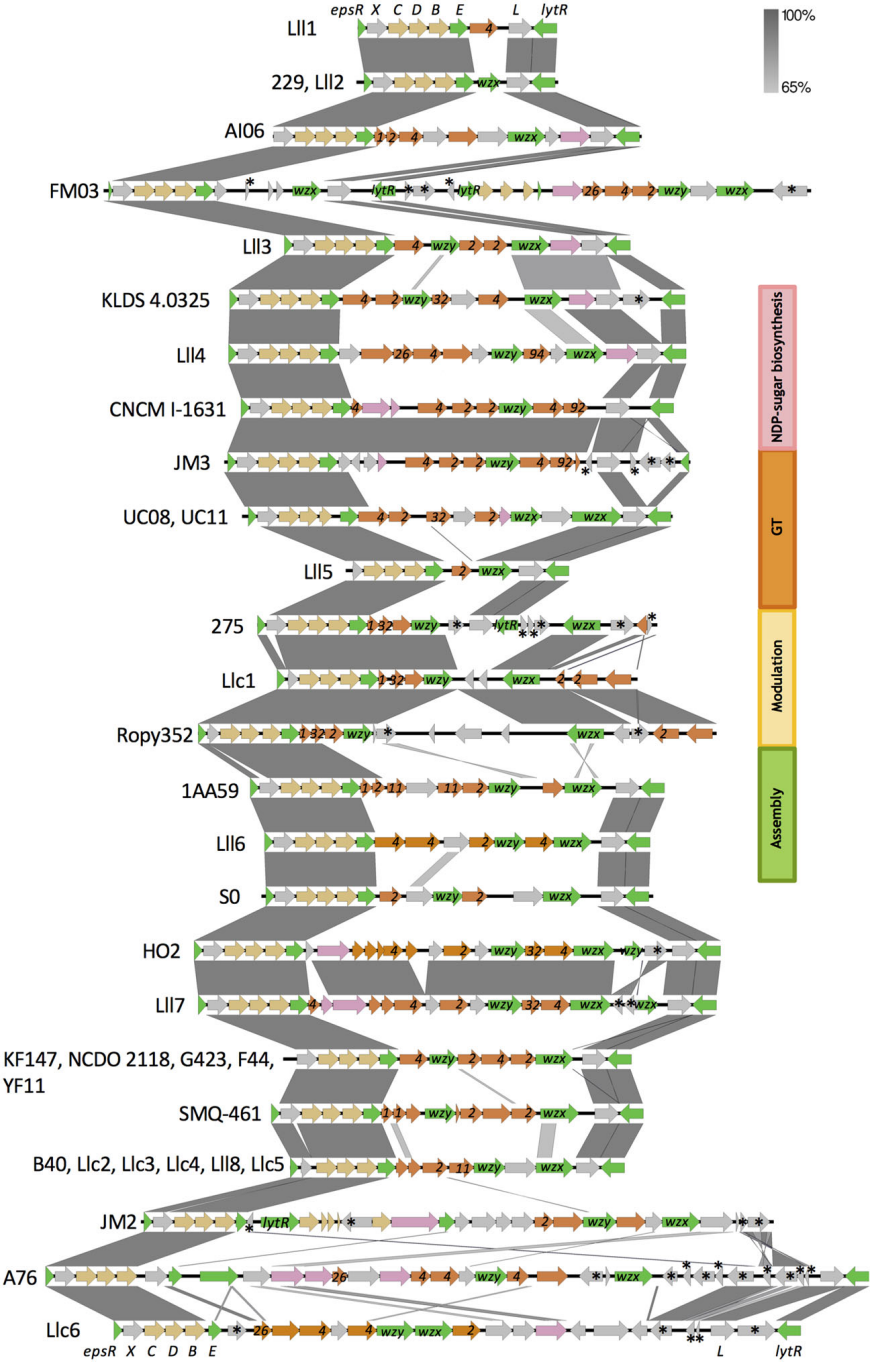
Comparison of *eps* gene clusters of selected *L. lactis* strains from the Chr. Hansen culture collection, as in Figs 1 and 2, and publicly available *L. lactis* genomes, 22 from the subsp. *lactis* and 13 from the subsp. *cremoris*; strain names are as in Figs 1 and 2. Genes in the *eps* operon were categorised into groups based on the putative or established functions of their products as in Zeidan *et al.* (2017). These include modulatory genes (yellow; phosphoregulatory module *epsCDB*), polysaccharide assembly machinery genes (green; initiation *epsE*, polymerisation *wzy*, export/flippase *wzx* and attachment *lytR*), genes encoding GT (orange; glucosyl transferases) necessary for the assembly of the repeating units, and genes encoding non-housekeeping functions (pink) required for the synthesis of activated sugar precursors and modification of the sugar residues. The functions of the three genes typically only present in the lactococcal *eps* gene clusters, *epsR, epsX* and *epsL*, remain to be elucidated. *Eps* gene cluster regions with 65%–100% identity are indicated with grey connection bars. Mobile genetic elements are marked with stars. Genes with unknown functions or functions that might not be related to the polysaccharide production, e.g. mobile genetic elements, are in light grey. Glucosyltransferases were marked with GT group numbers according to CAZy (database of Carbohydrate-Active enZYmes) classification, e.g. 2 means GT2 group. Abbreviations: GT, glycosyltransferase; wzy, polymerase; wzx, flippase; NDP-sugar, nucleotide diphospho-sugar.

A large diversity of genes encoding the synthesis of exocellular polysaccharides via the Wzy-dependent pathway were found in the genomes of *L. lactis* (Fig. 3). Here, we used the nomenclature suggested by Zeidan *et al.* (2017). The conserved genes in the beginning of the *eps* gene cluster were denominated *epsRXCDB*, and those at the end, *epsL* and *lytR*, while the polymerase was named *wzy*, and the flippase, *wzx* (Fig. 3). The organisation of the genes in the *L. lactis eps* clusters is similar to other LAB, but there are some distinguishing features. *LytR*, which is likely involved in the attachment of polysaccharides to the cell wall in LAB (Zeidan *et al.*^2017^), is the first gene of the cluster in most LAB (usually denominated *epsA* or *cpsA*) and in *Streptococcus pneumoniae*, while in *L. lactis* it is situated at the end of the *eps* gene clusters (Fig. 3). The first gene of the lactococcal *eps* gene cluster, *epsR*, is a regulator belonging to XRE family of proteins, which is not present in the *eps* gene clusters of other LAB (Zeidan *et al.*^2017^), indicating that *L. lactis* has a different way or an additional mechanism of polysaccharide biosynthesis regulation compared to other LAB.

Genes located at the 5' end of the *eps* gene cluster *epsRXCDB*, which are involved in the modulation and assembly machinery of polysaccharide biosynthesis, as well as *epsL* and *lytR* at the 3' end, displayed the highest level of conservation. The genes of the variable part, including polymerase *wzy*, flippase *wzx* and glucosyltransferases (GT) or other polymer-modifying enzymes, were rarely similar between the strains (Fig. 3), in agreement with what is observed in other organisms (Bentley *et al.*^2006^; Zeidan *et al.*^2017^). Based on CAZy (database of Carbohydrate-Active enZYmes) classification, one third of GT found in the *eps* gene clusters of the selected strains belong to GT2, one third to GT4 and one third to other CAZy groups (Fig. 3).

Among the non-texturing lactococcal strains from Fig. 2, Llc7 and Lll9 did not contain an *eps* gene cluster. Strains Llc1, Lll1, Lll2, Lll4, Lll5, Lll6 and Lll7 all contained an *eps* gene cluster. Three (Lll1, Lll2, Lll5) out of seven *eps* gene cluster positive but non-texturing strains contained presumably non-functional *eps* gene clusters. Lll1 had neither a putative polymerase *wzy* nor a flippase *wzx*; Lll2 was lacking both a polymerase *wzy* and GT; Lll5 had no polymerase *wzy* (Fig. 3). The four remaining non-texturing strains contained presumably functional *eps* gene clusters. However, it is not known if all the genes are functional. Even if strains can produce polysaccharides, they may not lead to texture in fermented milk. Because the presence of *eps* genes per se does not indicate whether a strain will contribute with texture in a particular food matrix, it is of importance to have a screening method that allows to detect strains with the desired texturing phenotype. This is achieved with the TADM screening method.

Based on *eps* gene cluster similarities, the eight texturing strains found in this study seem to fall into four different groups. Llc2, Llc3, Llc4, Llc5 and Lll8 all resemble NIZO B40, whereas strains Lll3, Llc6 and Ropy352 all have unique *eps* gene clusters (Fig. 3). All *eps* gene clusters have high similarity within the conserved regions (*epsRXCDBE*, *epsL* and *lytR*), but the remaining part of the *eps* gene clusters, including *wzy* (polymerase), *wzx* (flippase) and the GT genes were indeed variable, both in terms of sequence and in terms of number of genes present (Fig. 3). The *eps* gene clusters of the strains from the four groups (NIZO B40 vs Lll3 vs Llc6 vs Ropy352) were 61%–78% identical, based on the nucleotide sequences of the complete *eps* gene clusters, while their identity in the variable part was between 48 and 59%. Lll3 has three GT, while the NIZO B40-like strains have four GT and Ropy352 and Llc6 have five GT (Fig. 3). We speculate this may lead to polysaccharides with different repeating units. The common denominator for the texturing strains is that they all contain the genes required for the polysaccharide production, e.g. *epsCDBE-wzy-wzx* and GT (Zeidan *et al.*^2017^).

Several texturing strains from the subsp. *cremoris* have been reported, e.g. NIZO B40, SMQ-461, Ropy352, JFR1 (van Kranenburg *et al.*^1997^; Dabour and LaPointe 2005; Knoshaug, Ahlgren and Trempy 2007; Ayala-Hernández *et al.*^2008^). We report the first texturing strains from the subsp. *lactis*, Lll3 and Lll8; the species identity of both strains was confirmed using 16S. Lll3 has a unique *eps* gene cluster including 14 open reading frames covering 13 kb, which contains three novel GT. All three predicted GT gene products of Lll3 showed low amino acid similarity (up to 51% identity) with known GT. The three GT together with a putative nucleotide sugar dehydrogenase are potentially involved in sequential building of the repeating unit, although their specific functions and therefore order of action have not been demonstrated. Lll8 seems to have acquired the *eps* gene cluster plasmid typically found in the subsp. *cremoris* such as NIZO B40 strain, but also in several proprietary strains from the subsp. *cremoris*, where the *eps* gene clusters differ from each other by several nucleotides.

More research is needed to link *eps* genes directly with functional properties of fermented milk, such as texture. A study of the structure, molecular weight and amount of polysaccharides resulting from the *eps* gene clusters is necessary to establish such a link. A combination of two activities, linking polysaccharide gene clusters with polysaccharide structures, e.g. as it was done with *S. pneumoniae* capsular polysaccharides (Aanensen *et al.*^2007^), and linking polysaccharide structures with their functionality in the desired matrix, e.g. milk, might reveal the direct link between polysaccharide genes and functionality of the polysaccharides. This might enable prediction of the texturing properties of the strains based on their genome sequences.

## CONCLUSION

The present work describes a high-throughput screening method for texturing strains, which requires only small sample volume, e.g. 500 μl, and is significantly faster compared to the standard texture measurement by a rheometer. The method is useful when it comes to screening for microorganisms that are suitable for obtaining specific texturing properties of the milk gel. It also provides a tool for determining the impact of milk additives on their ability to impact shear stress of milk gels. The TADM-based high-throughput method allowed us to rapidly screen for novel texturing lactococcal strains suitable for use in preparation of mesophilic food products.

Comparative genomics of polysaccharide gene clusters of publicly available and proprietary texturing and non-texturing *L. lactis* strains revealed four different groups of texturing strains, based on their *eps* gene clusters. The two strains Lll3 and Lll8 are the first *L. lactis* subsp. *lactis* reported to enhance texture in fermented milk. Strain Lll3 has a novel *eps* gene cluster, whereas strain Lll8 has an *eps* gene cluster similar to that in NIZO B40. Texturing strains from the four groups are presumably producing different types of polysaccharides, as they have variable GT genes along with different *wzy* and *wzx* genes.
